# Genetic Diversity and Structure among Isolated Populations of the Endangered Gees Golden Langur in Assam, India

**DOI:** 10.1371/journal.pone.0161866

**Published:** 2016-08-26

**Authors:** Muthuvarmadam S. Ram, Sagar M. Kittur, Jihosuo Biswas, Sudipta Nag, Joydeep Shil, Govindhaswamy Umapathy

**Affiliations:** 1 Laboratory for the Conservation of Endangered Species, CSIR-Centre for Cellular and Molecular Biology, Uppal Road, Hyderabad 500007, India; 2 Primate Research Centre NE India, H/N 4, Byelane 3, Ananda Nagar, Pandu, Guwahati 781012, India; 3 Sálim Ali Centre for Ornithology and Natural History, Anaikatty, Coimbatore 641108, India; National Cheng Kung University, TAIWAN

## Abstract

Gee’s golden langur *(Trachypithecus geei)* is an endangered colobine primate, endemic to the semi-evergreen and mixed-deciduous forests of Indo-Bhutan border. During the last few decades, extensive fragmentation has caused severe population decline and local extinction of golden langur from several fragments. However, no studies are available on the impact of habitat fragmentation and the genetic diversity of golden langur in the fragmented habitats. The present study aimed to estimate the genetic diversity in the Indian population of golden langur. We sequenced and analyzed around 500 bases of the mitochondrial DNA (mtDNA) hypervariable region-I from 59 fecal samples of wild langur collected from nine forest fragments. Overall, genetic diversity was high (*h* = 0.934, *π* = 0.0244) and comparable with other colobines. Populations in smaller fragments showed lower nucleotide diversity compared to the larger forest fragments. The median-joining network of haplotypes revealed a genetic structure that corresponded with the geographical distribution. The Aie and Champabati Rivers were found to be a barrier to gene flow between golden langur populations. In addition, it also established that *T*. *geei* is monophyletic but revealed possible hybridization with capped langur, *T*. *pileatus*, in the wild. It is hoped that these findings would result in a more scientific approach towards managing the fragmented populations of this enigmatic species.

## Introduction

Gee’s golden langur *(Trachypithecus geei*), a colobine primate endemic to Indo-Bhutan border, was discovered in the late 1950s based on morphological differences with the capped langur (*T*. *pileatus*) [1,2]. It has been listed as endangered species in the IUCN Red List [3] and Schedule-I species in Indian Wildlife Protection Act (1972). Golden langur is found in the sub-tropical, monsoon-fed, semi-evergreen and mixed deciduous forests of western Assam in India and south-central Bhutan. It is considered to be one of the most restricted-range primates of South Asia [4–6]. Its distribution in India is confined between the rivers Manas in the east, Sankosh in the west, Brahmaputra in the south and high mountain ridges upto 2300 m altitude in the north, and in Bhutan it is found between Sankosh-Chamke-Mangde-Manas river complex [4–11]. The golden langur shares its habitat with other non-human primates such as the slow loris (*Nycticebus bengalensis*), Assamese macaque (*Macaca assamensis*) and rhesus macaque (*Macaca mulatta*). However, its distribution range rarely overlaps with that of the capped langur (*Trachypithecus pileatus)* but hybrids between golden and capped langur have been reported in Bhutan [11].

In Bhutan, the golden langur is mostly found in three Protected Areas viz. Black Mountain National Park (NP) (1,730 km^2^), Royal Manas NP (1,033 km^2^) and Phibsoo Wildlife Sanctuary (WLS) (266 km^2^) [12], whereas in India, except Manas NP (500 km^2^) and Chakrashila WLS (45.58 Km^2^), the major populations are found in Reserve Forests (RFs), Proposed Reserve Forests (PRFs) and Unclassified State Forests with a little or no protection. In the last few decades due to forest fragmentation and degradation, its habitat in India has been reduced by more than 30% leading to severe population fragmentation [4,5]. Of the estimated 6,500 individuals of Indian populations, 93% occur in three large Reserve Forests (Chirang, Manas and Ripu) and the western part of Manas NP, and the remaining occur in several small isolated fragments [13–17]. Recently, local population extinctions have been reported from seven isolated fragments [6].

Habitat fragmentation consistently has large, negative effects on measures of biodiversity such as population size, distribution and genetic diversity, especially in habitat specialist species [18–22]. Low genetic variation, in turn, results in reduction of survival and reproduction [23,24] which increases the probability of extinction [25–27]. To estimate genetic variation and structure within wild populations of primates, many studies have focussed on the use of mtDNA control region (D-loop), particularly the hypervariable regions I and II (HVRI and HVRII) because of their high rate of mutation and resistance to selection pressures [28–40]. Moreover, non-invasive samples such as feces and hair usually yield low-quality DNA and working with mtDNA is easier and more reliable due to their availability in high copy number in cells. The hypervariable region has also been used to study the evolution of pelage coloration in colobine species [41].

Genetic data generated so far on golden langur was intended to examine its taxonomic placement within the langur group in the subfamily Colobinae using mitochondrial cytochrome b gene (cytb) [10,42,43]. Interestingly, none of the above studies has used samples from wild populations of golden langur in India. Its placement in the colobine tree is of particular importance because of its presence where the ranges of the genera *Semnopithecus* and *Trachypithecus* meet. Furthermore, no information is available on the genetic diversity of fragmented populations of golden langur. The present study aims to estimate the genetic diversity in the Indian populations of golden langur using HVRI. It also aims to examine the phylogenetic position of Indian golden langur with respect to the Bhutanese population and closely related *Trachypithecus* spp. using cytb. It is hoped that this study would serve as a platform for a more scientific approach towards managing the wild population of this enigmatic species.

## Methods

### Study area

**Fig 1:**
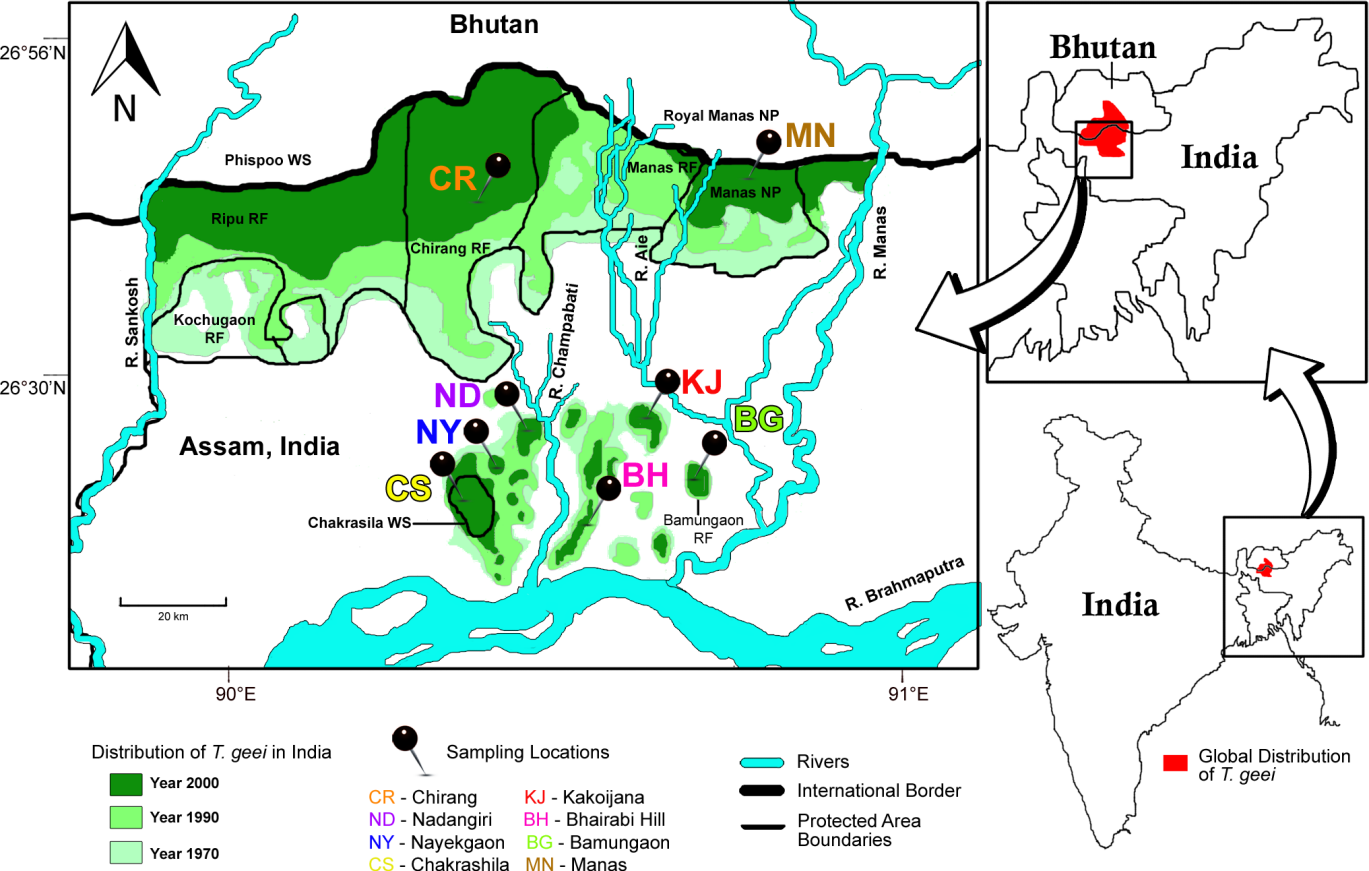
Study area and sampling locations. Current and past distribution of golden langur in Assam, India based on previous reports [4] and sampling locations in the present study. Umananda (UM) is not shown in this map. Modified and reprinted from Choudhury *et al* 2002 [4] under a CC BY license, with permission from Zoos’ Print Journal, Coimbatore, India, original copyright 2002.

Eight forest fragments in Assam that vary in size, time since isolation and disturbance level (Table 1) were part of the study, which include Chirang RF (CR), Manas NP (MN), Chakrashila WLS (CS), Bamungaon PRF (BG), Nayekgaon PRF (NY), Nadangiri RF (ND), Bhairabi Hill PRF (BH) and Kakoijana RF (KJ) (Fig 1). The fragment size ranged from eight km^2^ (NY) to 592.5 km^2^ (CR) and are inhabited by as few as 25 golden langur individuals (BG) to more than 1450 individuals (CR) [13–15]. Apart from these eight forest fragments, the study included the temple group in Umananda (UM), a small river island in Brahmaputra River which lies outside the natural range of golden langur. The temple group was founded by two individuals that are believed to be brought from Bhutan [17,44].

**Table 1:** Study sites and their isolation status.

Sl no.	Fragment	Area (km^2^)	GPS Coordinates	Population	Isolation status	Disturbance
1	Chirang (CR)	592.5	26°37’39.4”N 90°17’21.5”E	>1450	Isolated since 1990s after losing about 30% of the habitat.	Little or no disturbance
2	Manas (MN)	352	26°47’26.2”N 90°57’27.7”E	>200	Isolated since 1990s and is contiguous with Royal Manas NP, Bhutan.	Little or no disturbance
3	Chakrashila (CS)	45.6	26°16’50.7”N 90°20’44.1”E	>500	Isolated since 1990s from adjoining Nadangiri and Nayekgaon PRF	Felling in the fringe areas
4	Bhairabi Hill (BH)	36	26°18’27.8”N 90°32’06.6”E	26	Isolated since 1970s from adjoining Nakkati RF	Heavily degraded
5	Kakoijana (KJ)	17	26°25’41.9”N 90°39’15.9”E	144	Isolated since 1970s	Heavily degraded
6	Bamungaon (BG)	10.5	26°21’34.8”N 90°40’16.7”E	25	Isolated since 1970s	Heavily degraded
7	Nadangiri (ND)	10.2	26°25’08.5”N 90°21’36.0”E	>20	Isolated since 1990s from adjoining Nayekgaon PRF and Chakrashila	Partly degraded
8	Nayekgaon (NY)	<8	26°21’49.3”N 90°23’10.3”E	112	Isolated since 1990s from adjoining Nadangiri and Chakrashila	Partly degraded
9	Umananda (UM)	0.049	26°11’47.8”N 91°44’43.4”E	7	Founder individuals introduced in 1993	-

### Sample Collection

Sample collection was performed without direct interaction with the golden langurs and without causing any disturbance to their habitat. Samples were collected only from government-owned lands. Permission to collect fecal samples from Assam state were obtained from the Principal Chief Conservator of Forests (Wildlife), Assam Forest Department (Order No. 336, dated 16^th^ March, 2013) and Assam State Biodiversity Board (Letter No. ABB/Permission/2012/469, dated 31^st^ August 2015). Fresh fecal samples were collected between 29/06/2014 and 16/12/2015 from wild langurs after watching them defecate. Care was taken not to collect multiple samples from the same individual. A total of 71 samples were collected from CR (N = 15), CS (N = 12), KJ (N = 10), ND (N = 9), NY (N = 6), BH (N = 6), UM (N = 5), MN (N = 4) and BG (N = 4). Approximately 5g of the samples were stored in small plastic bottles using the two-step method [39]. In short, the samples were first soaked in 90% ethanol for 24 hours, dried, stored with silica gel and transported to the lab as soon as possible.

### DNA isolation, amplification and sequencing

Fecal material was dried overnight in hot air oven at 50°C to remove any moisture. 0.2g of the completely dried fecal material was scrapped from the surface and used for extraction. Genomic DNA was extracted from the samples using Qiagen Stool Kit following the manufacturer’s protocol. The extracted DNA was stored in the elution buffer provided in the extraction kit. DNA quantification was done using Nanodrop-Spectrometer.

To avoid amplification of nuclear inserts of mitochondrial DNA fragments (numts), a PCR was performed with universal vertebrate mitochondrial cytochrome b (cytb) forward primer [45] and 16022R reverse primer [46] which generated a 1500 bp PCR product. A nested PCR with PresLoopF [46] and 16220R primers was then performed, which generated PCR products of 690 bp of partial control region, specifically the hypervariable region-I (HVRI), partial tRNA-Thr, and complete tRNA-Pro. Additionally, 450 bp of partial cytb was also amplified using the universal cytb primers [45].

PCRs were carried out with initial denaturation at 95°C for 5 min, 35 cycles each with initial denaturation at 95°C for 20 sec, annealing at 52°C for 30 sec and extension at 72°C for 30 sec followed by final extension at 72°C for 10 min. The total reaction volume was 15 μl with 1X BSA, 1X PCR buffer, 0.25 mM of each DNTP, 2.5 mM MgCl_2_, 0.25 μM each of forward and reverse primers and 0.75 units of Taq polymerase (ExTaq HS DNA polymerase, Takara Bio Inc.). Utmost precautions were taken while performing PCR to avoid contamination from an external source of DNA. All PCR reactions were carried out with a negative control. Pre- and post-PCR works were carried out at separate places and using separate micropipettes. The PCR products were visualised on a 2% agarose gel. DNA was eluted from the gel using Gel Elution Kit (Bioserve India).

DNA sequencing was carried out using BigDye Terminator cycle sequencing Kit (Applied Biosystems) in ABI 3730XL sequencer. Sequencing was repeated twice in both the directions. A maximum 639 bases of a mitochondrial DNA fragment containing the partial tRNA-Thr sequence, complete tRNA-Pro sequence and 543 bases of HVRI were generated. Cytb sequences of a maximum 521 bases were generated from eight samples.

### Genetic analysis

The sequences were assembled and manually edited to check for base-calling errors using CodonCode Aligner v.2.0.6 (http://www.codoncode.com/). The primer sequences along with low quality bases at the ends which were removed. CLUSTAL W with default settings as implemented in MEGA5 was used to realign the edited sequences.

Diversity estimations were done using DnaSP v.5.10 [47]. Genetic diversity was estimated in terms of average haplotype diversity (*h*) and nucleotide diversity (*π*) for each fragment. Population pairwise F_ST_ were calculated from pairwise nucleotide differences between fragments and their statistical significance was checked using 10,000 permutations as implemented in Arlequin v.3.5.1.2 [48]. To represent the geographical distribution of the haplotypes and the mutational relationships of the haplotypes among the isolated populations, the software NETWORK v.5.0.0.0 (available at http://www.fluxus-engineering.com) was used to construct a median-joining haplotype network [49].

For determination of intra-specific genetic structure, all unique HVRI haplotype sequences of *T*. *geei* were used. Four orthologous sequences of other *Trachypithecus* spp. truncated from mitogenome sequences from GenBank nucleotide database were part of this 529 base dataset: two sequences of *T*. *shortridgei* (accession numbers HQ149048.1 and KP834334.1) and one sequence each of *T*. *pileatus* (acc. no. KF680163.1), and *T*. *obscurus* (acc. no. EU004477.1). Two sequences of *Nasalis larvatus* (acc. nos. DQ355298.1 and KM889667.1) were used as the outgroup. In addition to HVRI sequences, cytb sequences were used for ascertaining the phylogenetic position of Indian *T*. *geei* sequences with respect to *T*. *geei* from Bhutan, *T*. *pileatus* and *T*. *shortridgei*. For this purpose, apart from the aforementioned mitochondrial sequences, three partial cytb sequences of *T*. *geei* (acc. nos. EU519220.1, EU526384.1 and EU526385.1) and one of *T*. *pileatus* (acc. no. EU526386.1) of Bhutanese origin were also included in the analysis.

The software jModelTest v.2.1.3 [50] was used to determine the simplest model of sequence evolution that best explains the nucleotide variations in the dataset. HKY+G model was selected based on interpretation of likelihood scores using Bayesian Information Criterion and Decision Theory. Bayesian Inference and Maximum Likelihood trees were reconstructed using Mr.Bayes v.3.1.2 [51] and raxmlGUI [52], respectively. For Bayesian Inference tree reconstructions, two parallel MCMC runs of two million generations were performed with three heated chains and one cold chain each. Trees were sampled every 100 generations. The split frequency of standard deviation, that was calculated once every 1,000 generations, was used to assess the convergence of the two runs. The first 25% of generations were discarded as burn-in. The uncorrelated potential scale reduction factor (PSRF) values for all parameters were confirmed to be approximately equal to one. The trees were summarized to give a consensus tree with posterior probability values for each branch. Maximum Likelihood (ML) tree reconstruction was carried out in raxmlGUI with 10,000 rapid bootstrap replications using GTRGAMMA as the substitution model.

## Results

### Genetic diversity

The possibility of amplifying numts was greatly reduced by the fact the all our DNA was extracted from fecal samples, which are naturally enriched with mtDNA molecules due to their small size, high copy number and lower vulnerability to degradation compared to nuclear DNA [53]. Moreover, all our PCR products were of the expected length and no extremely variant sequences were detected. Furthermore, during an initial tree construction with all available *Trachypithecus* sequences in GenBank, our sequences clustered with authentic mtDNA sequences instead of confirmed pseudogenes. No stop codons were detected in the partial cytb sequences.

Out of 71 samples collected, 59 samples gave successful amplification for HVRI and 518 bases were sequenced and aligned. Nineteen haplotypes were revealed based on 47 mutations (45 transitions and two transversions) spread over 46 segregating sites, out of which 43 were parsimony-informative. Haplotype sequences were deposited to GenBank (accession numbers KX189494-KX189512).

The overall haplotype diversity was high (*h* = 0.938) but the nucleotide diversity was low (*π* = 0.02443). The haplotypes were assigned an alphabetical code (from GL-A to GL-S) based on the chronology of discovery. Haplotype GL-C was the most frequent, represented by eight individuals from two fragments, followed by GL-A represented by seven individuals. More than half of the haplotypes represented fewer than three individuals.

Haplotype and nucleotide diversities were calculated for each population (Table 2). CS and BH showed the highest number of haplotypes (four each) and CR showed the second highest (three haplotypes). Consequently, CS and BH also had the highest haplotype diversities (0.76 and 0.8 respectively). Five fragments (KJ, NY, ND, MN and BG) showed two haplotypes while UM showed no variation and KJ, ND and BG showed the least number of polymorphic sites among their haplotypes. Surprisingly, NY, which had only two haplotypes, showed the most number of polymorphic sites (17) for any fragment, giving it the highest nucleotide diversity. It was followed by CR (14), CS (7), MN (7) and BH (6). KJ had the lowest nucleotide diversity (*π* = 0.000483).

**Table 2:** Fragment-wise estimates of genetic diversity in the golden langur.

Sl. no.	Fragment name	Samples collected	Sequences analyzed	Haplotypes	Polymorphic sites	Haplotype diversity *h* (SD)	Nucleotide diversity *π* (SD)
1	Chirang (CR)	9	7	3	14	0.524 (0.209)	0.00919 (0.00436)
2	Manas (MN)	4	4	2	7	0.5 (0.265)	0.00676 (0.00517)
3	Chakrashila (CS)	12	11	4	7	0.764 (0.099)	0.00527 (0.0007)
4	Bhairabi Hill (BH)	6	6	4	6	0.8 (0.172)	0.00541 (0.00156)
5	Kakoijana (KJ)	10	8	2	1	0.25 (0.18)	0.00048 (0.00035)
6	Bamungaon (BG)	4	3	2	1	0.667 (0.314)	0.00129 (0.00061)
7	Nadangiri (ND)	9	5	2	1	0.6 (0.175)	0.00116 (0.00034)
8	Nayekgaon (NY)	6	6	2	17	0.6 (0.0129)	0.01969 (0.00424)
9	Umananda (UM)	5	5	1	0	0	0

### Genetic structure

**Fig 2:**
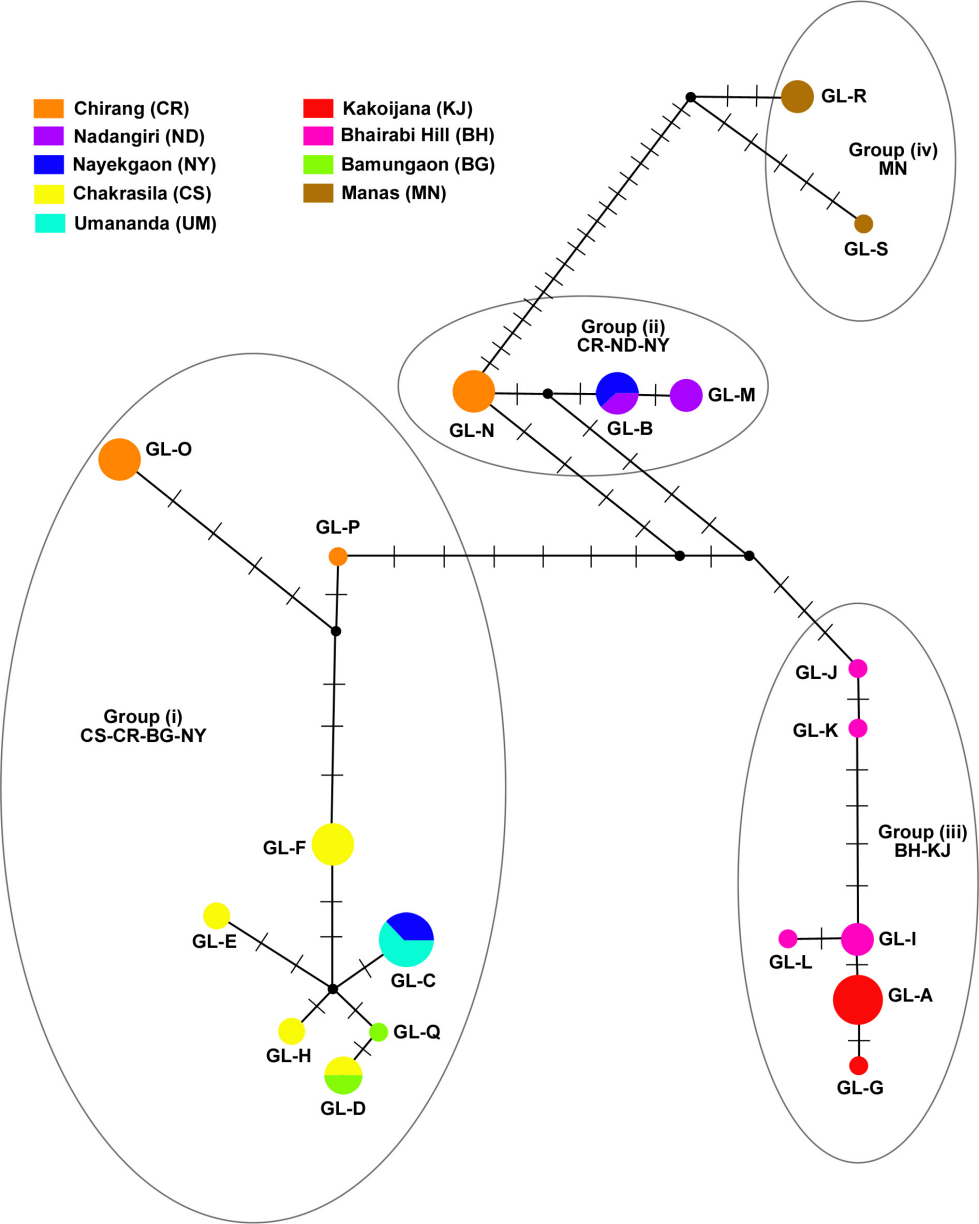
Median-joining network. Median-joining network of mtDNA HVRI haplotypes of golden langurs. The size of each circle represents the frequency of each haplotype (N = 59) and the colours represent each fragment. Each bar on the lines connecting two haplotypes represents one mutational step.

A median-joining haplotype network was drawn based on the HVRI sequence data (Fig 2). The 19 haplotypes differed from each other by between 1 and 42 bases. Three out of the 19 haplotypes were shared between utmost two fragments- haplotype GL-B was shared between NY and ND, GL-C was shared between NY and UM, and GL-D was shared between CS and BG. The haplotype network revealed no clear-cut structure, but there are at least four distinct groups- (i) all CS haplotypes along with two out of the three CR haplotypes and two other haplotypes from BG and NY/UM, (ii) Two ND haplotypes and the remaining CR haplotype, (iii) all BH and KJ haplotypes and (iv) all MN haplotypes (Fig 2).

The pairwise F_ST_ were calculated between each fragment and statistical significance assessed with 10,000 permutation steps (Table 3). NY was not significantly different from ND, UM and CR, and CS and BG were not significantly different from each other. Of the groups that were significantly different, NY and BG were most similar, followed by CS and UM, and CS and NY. The most dissimilar groups were KJ and UM.

**Table 3:** Pairwise F_ST_ values between fragments (below diagonal) and their significance (above diagonal) after 10,000 permutations.

	CR	MN	CS	BH	KJ	BG	ND	NY	UM
CR	0	+	+	+	+	+	+	-	+
MN	0.76278	0	+	+	+	+	+	+	+
CS	0.54884	0.90687	0	+	+	-	+	+	+
BH	0.51405	0.89296	0.81496	0	+	+	+	+	+
KJ	0.67172	0.95875	0.87668	0.50222	0	+	+	+	+
BG	0.52859	0.92501	0.27654	0.89701	0.97673	0	+	+	+
ND	0.45262	0.92393	0.86979	0.84401	0.97244	0.95869	0	-	+
NY	0.1784	0.72321	0.40284	0.5	0.66239	0.27427	0.32999	0	-
UM	0.60446	0.95098	0.39934	0.89701	0.98876	0.91477	0.98171	0.3617	0

‘+’ indicates significant F_ST_ (p<0.05) and ‘-’ indicates not significant.

**Fig 3:**
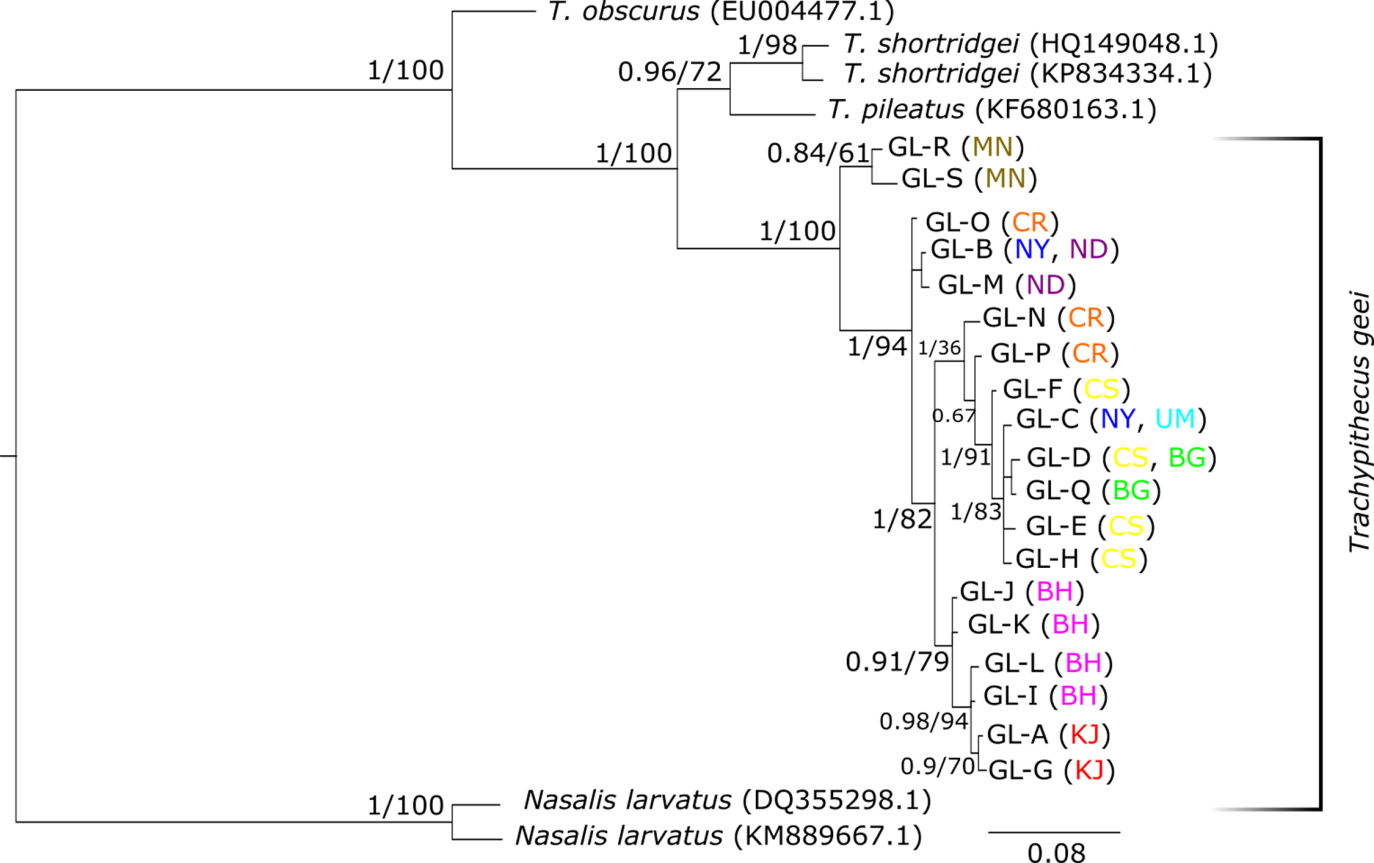
Bayesian Inference tree of hypervariable region-I. Bayesian inference tree reconstructed from 518 bases of HVRI. Leaves are labelled with the species and accession codes in parentheses and, for golden langurs, haplotype codes followed by the fragment(s) of origin in parentheses. The numbers at each node are the Bayesian posterior probabilities followed by bootstrap values of the corresponding nodes (where present) in the maximum likelihood tree.

The 19 HVRI haplotypes of *T*. *geei* along with GenBank sequences of *T*. *pileatus*, *T*. *shortridgei* and *T*. *obscurus*, and *Nasalis larvatus* as the outgroup were used to reconstruct phylogenetic trees. Both Bayesian Inference and Maximum Likelihood trees gave almost the same tree topology and similar statistical support for most clades (Fig 3). In both trees, *T*. *geei* emerged as a monophyletic clade with very high posterior probability/bootstrap value and showed most recent divergence from the lineage leading to *T*. *pileatus* and *T*. *shortridgei*. Golden langurs fell into two distinct clades, one containing the two haplotypes from MN and the other containing all the remaining haplotypes. Both these clades were statistically well-supported. In the second clade, haplotypes GL-B (NY and ND) and GL-M (ND) were basal to the rest of the 15 haplotypes of which 14 fell into two clades of high statistical support, and one (GL-O from CR) remained unresolved. The BH-KJ clade showed a typical stepping stone-like progression from BH haplotypes to the KJ haplotypes, with high support for all nodes but one. On the other hand, the CR-CS clade also showed a similar progression from CR haplotypes to the rest. However, the basal node had low support and the derived sequences fanned out instead of being strictly dichotomous, but the nodes had high support.

**Fig 4:**
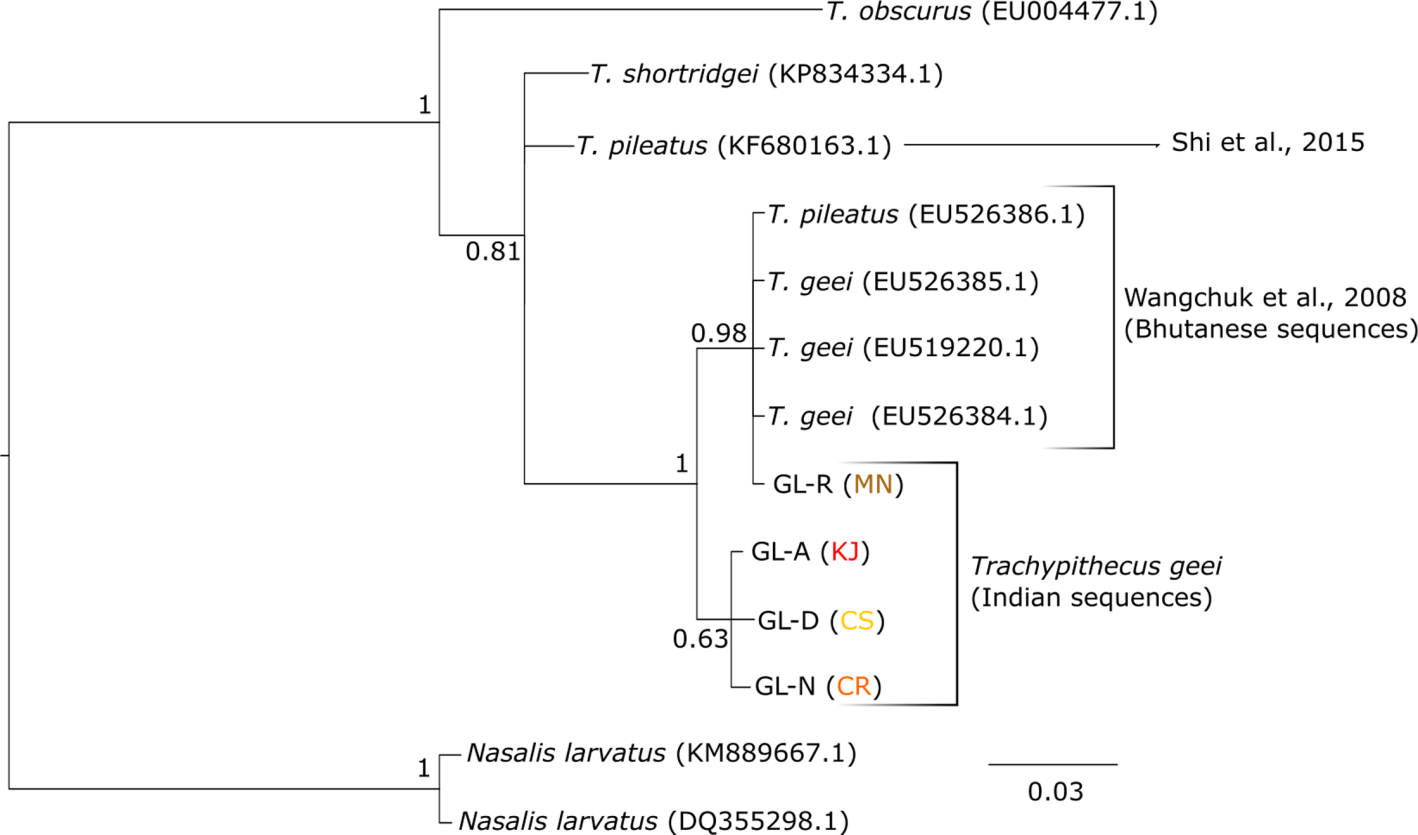
Bayesian Inference tree of cytochrome b. Bayesian inference tree reconstructed from 392 bases of cytb. Leaves are labelled with the species and accession codes in parentheses and, for golden langurs, haplotype codes. The numbers at each node are the Bayesian posterior probability values. Sequences generated in the present study and by other authors have been indicated.

We also found that the cytb sequences obtained from *T*. *geei* formed four haplotypes (named GL-A, GL-D, GL-N and GL-R, according to their corresponding HVRI haplotypes). Haplotype sequences were deposited to GenBank (accession numbers KX189513-KX189516). To check for the taxonomic placement of *T*. *geei*, a dataset containing 392 bases of partial cytb sequences was used in phylogenetic tree reconstruction. *T*. *geei*, *T*. *pileatus* and *T*. *shortridgei* clustered together with very high support, as expected, but the relationship among the three remained unresolved (Fig 4). Our sequences of *T*. *geei* clustered with the three sequences of Bhutanese *T*. *geei*. The haplotype GL-R from Manas had the same sequence as the Bhutanese *T*. *geei*. Curiously, one of the *T*. *pileatus* sequences (sampled from Bhutan by Wangchuk et al., 2008 [43]) also clustered with this *T*. *geei* clade (posterior probability of 1), whereas the other *T*. *pileatus* sequence [54] separated out.

## Discussion

### Genetic diversity in the fragmented forests

While all previous genetic work carried out on golden langur were aimed at addressing questions regarding its taxonomic placement among congeneric species [10,42,43], the present study aimed to estimate its genetic diversity. To achieve this, 59 golden langur individuals were sampled from nine sites of varying sizes and times since isolation, and a 518 base segment of HVRI was sequenced. Golden langur showed a very high haplotype diversity (*h* = 0.938) and a moderately high nucleotide diversity (*π* = 0.0244) compared to other Asian colobines. For example, the critically endangered white-headed langur, *T*. *leucocephalus* (*h* = 0.57, *π* = 0.00323) and Guizhou snub-nosed monkey, *Rhinopithecus brelichi* (*h* = 0.457, *π* = 0.014) showed much less genetic diversity even when a much higher percentage of their population was sampled [36,40]. These species have severely depleted populations due to habitat loss and have an extremely small geographical distribution. In spite of golden langur’s small geographical footprint, its overall genetic diversity is comparable to other endangered Asian colobines with wider distribution like Sichuan snub-nosed monkey, *R*. *roxellana* (*h* = 0.845, *π* = 0.034) [55], Yunnan snub-nosed monkey, *R*. *bieti* (*h* = 0.944, *π* = 0.034) [31,56] and proboscis monkey, *Nasalis larvatus* (*h =* 0.9, *π* = 0.022) [35]. Notably though, the nucleotide diversity in golden langur is comparatively low, perhaps because of a more recent divergence from its sister species compared to *Rhinopithecus* spp. [36,43].

Golden langur, once found in large populations, now occurs in several isolated populations. The highest haplotype diversities were observed in Bhairabi Hill and Chakrashila fragments (30–50 km^2^), whereas lowest was in Kakoijana (10 km^2^). Interestingly two of the largest (>350 km^2^) fragments showed lower haplotype diversities. This might be due to over-sampling of major haplotypes. Nucleotide diversities showed greater correspondence to the fragment areas with larger fragments showing higher nucleotide diversities compared to smaller fragments, with the exception of Nayekgaon, thus reflecting the greater gene flow expected in large, continuous forests. Nayekgaon’s unusually high nucleotide diversity may be due to the presence of individuals from divergent populations which originated from adjoining fragments which it was connected with until the 1990s. Kakoijana, although not the smallest fragment, has the lowest haplotype and nucleotide diversities, which could be due to relatively large geographical and temporal isolation from adjoining forest (Fig 1, Table 1). Umananda population, being very recently founded by two individuals that were donated by devotees, showed no variation. Overall nucleotide diversity increased with increases of population and area of the fragment, while haplotype diversity did not, however more samples from more groups in larger fragments are required to ascertain the relationship.

### Genetic structure in the golden langur

To examine the relationship among fragmented populations, we constructed a median-joining haplotype network (Fig 2) and phylogenetic trees (Fig 3) using HVRI haplotypes. The haplotypes fall broadly into four groups and two of them (MN and BH-KJ) are well-defined. The Aie River, one of the largest rivers to occur in the distributional range of golden langur (Fig 1), separates the Manas RF and Manas NP from the other forests. Therefore, it should not come as a surprise that haplotypes from MN are the most distant. The perennial Champabati River (Fig 1) also creates a barrier to westward movement of golden langurs from BH and KJ, meaning that any movement from this area to CS, NY, ND or CR can be achieved only by circumventing it. The UM temple group shared its haplotype with CS indicating that the founder individuals were from CS. In general, the haplotype network corresponded well with the geographical origins of the samples. However, there were some discrepancies. Firstly, NY contains two haplotypes that differ by a massive 17 nucleotide bases. While one of these is shared with ND and falls in CR group, the other is shared with UM and falls in the CS group (Fig 2). This is evidence of past connectivity of CR with NY, ND and CS and gene flow among them. This argument is bolstered by the fact that there are no natural barriers that occur between CS-NY-ND and CR. It is likely, therefore, that recent anthropogenic modification of the habitat has isolated CS-NY-ND from CR. Secondly, BG which is the easternmost small isolated fragment shares haplotypes with the distant CS fragment. This might be due to introduction of individuals from CS, but this needs further detailed investigation.

The phylogenetic trees reconstructed with HVRI haplotypes (Fig 3) revealed an early divergence of golden langur into two clades separated by the Aie River (MN to the east and the rest to the west of Aie). In addition, it was found that in the west clade, NY, ND and CR haplotypes were basal to the remaining 14 haplotypes which form a clade. The area encompassing NY, ND and CR could be considered as the putative place of origin of the west clade. In late 1960s the area was more or less interconnected with each other but now fragmented in to Gaourang RF, Nadangiri RF and Nayekgaon PRF. The barrier (most probably the Champabati River) between CS-NY-ND-CR and BH-KJ seems to have been formed after an early expansion of golden langurs from near ND. After the barrier was formed, there seems to have been a range expansion through migration from ND-CR to CS and from BH to KJ, as evidenced from the stepping-stone-like progression.

### Golden langur phylogeny and hybridization

Gee’s golden langur appears to be monophyletic with respect to its sister species (Fig 3). The cytb tree (Fig 4), however, throws some light on the possibility of hybridization with *T*. *pileatus*, especially in Bhutan. A previous report from Bhutan did not find sequence variation between golden and capped langur [43]. This might be due to sampling of hybrid individuals. Golden langur is often confused with capped langur owing to their variable coat morphology [1], seasonal variations in coat colour [2] and difficulty in observing the individuals through thick canopies. Moreover, natural hybrids between golden langur and capped langur have also been observed in their range peripheries [11]. Ironically, though, our study also supports Wangchuk *et al*.’s [43] conclusion that the golden and capped langur individuals of Indian zoos sampled by Karanth [42] are hybrids with *Semnopithecus entellus*. However, future studies should use nuclear markers to determine the genetic structure of the paternal component in golden langur.

### Management recommendations

Although a majority of the fragments showed moderate to high genetic diversity for small, isolated populations, some fragments showed very low genetic diversity, particularly Kakoijana which has a population of about 144 golden langurs. Kakoijana was connected to Nakkati and Bhairabi Hill until the 1970s. Steps should be taken to re-establish this link through canopy corridors to facilitate gene flow between Kakoijana and neighbouring fragments. The group in Bamungaon (25 individuals) seems to be founded by individuals from or near Chakrashila. This has to be ascertained to prevent any inadvertent genetic intermixing during future repopulations. Most forest fragments lost their connectivity between 1970s and 1990s. These fragments should be protected from further degradation and steps may be taken to connect them to maintain genetic diversity.

Golden langurs are monophyletic, and it appears that they are capable of hybridizing with capped langurs in the wild and Hanuman langurs in captivity. In fact, some of the individuals kept in Indian zoos may be hybrids. Therefore, the genetic affiliation of captive golden langurs has to be ascertained before they are used in captive breeding programs and reintroductions.
